# Supplementary material for: Are carbon emissions trading and green financial instruments synergistic? -Comprehensive quantitative research based on content analysis
Source: PLoS One. 2024 Mar 7;19(3):e0298601. doi: 10.1371/journal.pone.0298601 (PMC10919601; doi:10.1371/journal.pone.0298601)
Supplement: S1 Data — (ZIP) [file pone.0298601.s001.zip › Data/地级市Z_F工作报告.zip!/░▓╗╒/╗┤─╧╩╨/└┤╫╘╣1⁄2╓┌║┼í╛┬φ┐╦╩2╛▌═°í┐_2008.docx]

各位代表:
现在，我代表市人民政府，向大会报告工作，请予审议，并请市政协委员和其他列席人员提出意见。
一、关于2007年工作及五年来主要成就
2007年，是我市奋力崛起进程中极不平凡的一年，全市人民在市委的领导下，全面贯彻落实科学发展观，紧紧围绕以人为本的和谐社会建设，牢牢把握宏观调控带来的有利条件，努力克服国际国内复杂因素和洪涝灾害形成的影响，经济和社会各项事业保持了平稳健康快速协调发展的良好势头。
——国民经济平稳快速增长。预计全市实现生产总值355亿元，增长12.1%，其中第一产业增加值36亿元，增长2.8%，第二产业增加值197亿元，增长14.3%，第三产业增加值122亿元，增长11.2%；实现财政总收入48.5亿元，增长12.8%，其中地方财政收入23.1亿元，增长17.5%；全市金融机构各项存款余额450亿元、贷款余额365亿元，分别比年初增加49亿元和73亿元；固定资产投资243亿元，增长22.8%，为历史最好水平；全社会消费品零售总额109.0亿元，增长16.5%，提前实现“百亿商贸”目标。
——社会事业加快发展。人口自然增长率6.85‰；城镇登记失业率4.2%；高中阶段毛入学率66.3%；城乡低保覆盖率分别达6.9%和3.7%；亿元生产总值生产事故死亡率比上年下降14.29%。
——资源环境得到有效治理保护。初步测算，万元生产总值能耗下降4.2%；二氧化硫、化学需氧量排放分别下降6%和7%。
——人民生活有较大改善。预计城镇居民人均可支配收入1.2万元，增长12.5%；农民人均纯收入　　3600元，增长10.7%；居民消费价格总水平上涨5%，低于全省平均水平。
一年来，我们主要抓了以下几方面工作：
第一，贯彻落实宏观调控政策，经济运行质量进一步提高。着力解决经济运行中的突出矛盾和问题，努力在贯彻宏观调控中又好又快发展。认真落实各项支农惠农政策，大力促进农民增收，投入各项支农资金6.4亿元，增长23%。加快实施工业强市战略，新增规模以上工业企业15户，预计完成规模以上工业增加值162亿元，增长15.5%；企业经济效益综合指数151，提高10个百分点；实现利税41亿元，增长9.7%。着力优化投资结构，加工制造业完成投资13.5亿元，增长22%。进一步扩大消费需求，城乡市场持续活跃，旅游、金融等服务业迅速发展。严格土地审批和环境评价，强化节能减排措施，整顿规范小煤矿、山石开发秩序，关闭了一批小煤矿、小机组、小水泥、采石场、石灰窑，淘汰了一批落后生产能力。促进县区经济加快发展，4个区财政收入增幅超过20%，新增财政收入超亿元的区2个，凤台县继续位居全省十强县前列。
第二，奋力抗洪救灾，灾后重建和新农村建设进一步深入。面对淮河1954年以来最大的流域性洪水，坚持把人民群众生命安全放在首位，运用了4个行蓄洪区，组织各方面力量，调集大批物资，投入各类资金2亿余元，6026户倒房户已全部搬进了新居，实现“六有一无”目标，取得了抗洪救灾和灾后重建的全面胜利。扎实推进新农村建设，示范村镇建设和村庄整治取得积极成效。粮食生产克服严重洪涝灾害影响，总产132.3万吨，创历史新高。建成国家级农业标准化示范区1个，通过国家绿色食品认证12个、无公害农产品认证12个。加强农村公共事业，建成“万村千乡”市场工程农家店178个、农家书屋30个，新建农民体育健身工程12处，改扩建农村敬老院12所。加快推进采煤沉陷区治理，基本建成市矿统筹新农村建设试点刘龙新村。村村通工程、沼气工程、治淮工程取得新进展，完成农村公路建设315公里，完成农田水利土石方517.8万立方，新建沼气池4000口。顺利完成小岛移民迁建工程，彻底解决了853户小岛居民的水患之害和恶劣的居住环境，终于实现了小岛人近半个世纪的梦想。
第三，推进重大项目建设，产业结构进一步优化。新型能源基地建设取得突破性进展，顾桥、刘庄等6个现代化大型煤矿和改扩建矿井竣工投产或试运转，口孜东煤矿通过国家核准，新增煤炭产能2300万吨；田集电厂一期、平圩电厂二期、洛河电厂三期等技术起点较高的燃煤电厂相继建成发电，新增电力装机容量387万千瓦；新上了一批符合工业化要求的煤电项目和配套工程。加快建设安徽煤化工基地，强力启动煤化工新区及年产60万吨甲醇、20万吨二甲醚项目，煤基合成油催化剂、甲醇制丙烯工业试验装置等科技先导型项目开工建设，蓝天化工厂移址新建，德邦100万吨纯碱扩建一期竣工投产。规划建设了安徽煤机装备制造基地、省级生物医药及高新技术产业基地、绿色食品加工基地。万向二期、山河药用辅料、益益万吨配方奶粉等一批项目相继竣工。凯盛重工、万泰机械电子、光华光神特种线缆、电子产品制造基地等一批项目开工。资源综合利用取得新进展。新上了一批煤矸石发电厂、瓦斯和低温余热电站，新建新型墙体材料生产线9条。服务业水平进一步提升。苏宁、幸福树电器等知名企业落户淮南，家乐福购物广场、商之都淮南购物中心、华联商厦扩建等项目开工。新创国家3A级旅游景区1个、2A级1个，建成了一批“农家乐”项目。
第四，改革开放向纵深拓展，经济社会发展活力进一步增强。我市被列为中部老工业基地试点范围。增值税转型试点改革的实施，对增强企业造血能力、推进结构调整具有深远意义。国有资产管理体系和公共财政体系进一步健全，国企改革和金融改革进一步深化。全面完成国有企业清产核资和行政事业单位国有资产清查工作。实施改制重组企业8户，整合医院7家。全市不良金融资产打包处置工作全面启动。国投新集公司成功上市，实现上市企业“零”突破。全省首家（淮南矿业集团）财务公司开业。城投、国资等融资平台运作成效明显，全年实现融资50亿元，与中铁四局签定了45亿元山南新区投资开发合同。农村综合改革、集体林权制度改革进展顺利。行政管理改革迈出新步伐，出台了《部分行政管理权限下放县区管理的实施意见》，下放权限76项。行政审批事项继续清理，政务服务中心“一站式”水平提高。对外开放取得新成效，预计全年实际利用外资2亿美元，增长11.0%；实际到位省外资金88.0亿元，增长28.0%。预计完成外贸进出口总额9000万美元，增长14.5%。开发区建设取得新进展，全年新批入区项目25个，竣工项目17个。朝阳东路延伸段竣工，打开了“东扩”的通道。田集海关保税仓库揭牌运作。对外交流合作取得新突破，与合肥市签订了《加强区域合作的框架协议》。
第五，加快城市基础设施建设步伐，城市功能进一步完善。完成市矿统筹规划和2个分区规划、2个控制性规划、6个专项规划编制。交通设施建设取得新成绩，合淮阜高速公路淮阜段建成通车，淮蚌高速公路淮南段征地拆迁等前期工作完成。山南新区建设取得新突破，洞山隧道全面贯通；南纬三路一期、陈洞南路一期建成，北环路、高压走廊改造、三和农民新村等工程开工。城市基础设施得到加强。完成火车站站前广场及龙湖路南段、李嘴孜排污口改造；市体育文化中心建成投入使用；广场北路、淮河大桥改造、龙王沟泵站扩建、东部生活垃圾处理场、西部城区供水管网改造、城市集中供热改造、市消防指挥中心等工程开工建设。新购公交车50辆，建设天然气加气站3座。实施农贸市场升级改造项目10个，竣工9个。旧城改造继续推进，施工商品房面积490万平方米，竣工142万平方米。生态淮南建设取得重大进展，创建国家园林城市通过考核验收。泉大资源枯竭矿区生态修复一期工程实施，十涧湖国家城市湿地公园开工；新建、改造游园10处，成片造林2.4万亩。创建中国优秀旅游城市进展顺利，文明创建活动取得实效，基本建成中心城区图像监控系统，城市综合管理水平迈上新台阶。
第六，实施科教兴市和人才强市战略，各项社会事业发展进一步协调。列入国家、省科技攻关项目9项，完成省级科技成果鉴定33项，认定省级高新技术企业6家、产品6个，申请专利244件，新增省名牌产品4个。创新平台建设取得新进展，煤矿安全高效开采省部共建重点实验室、现代矿业省级重点实验室批准建设。大力推进义务教育均衡发展，改扩建农村中小学81所，改造D级危房2.4万平方米，选派510名教师到农村支教。加快发展高中阶段教育，新创省示范高中1所，安徽工贸职业技术学院被评为全国职业教育先进单位，中职招生人数增长30.5%。高等教育办学规模扩大、质量提高，安徽理工大学跻身全国高校本科教学评估优秀行列，淮南师院、淮南联大新校区加快建设。文化、体育事业得到加强。新四军纪念林等3个基地被命名为省级爱国主义教育基地，紫金砚等5个项目入选省首批非物质文化遗产保护名录，市博物馆二期工程全面实施。我市被中国烹饪协会授予“中国豆腐之乡”，凤台县被中国舞协授予“中国民族民间舞蹈艺术之乡”。成功举办第十四届中国豆腐文化节。中国文联、全国妇联、安徽省人民政府联合在淮南举办了首届国际少儿艺术节，25个国家和地区少年儿童代表参加了“5+1”的各种活动，淮南的少儿艺术开始真正走向世界。新闻出版工作得到加强，广播电视在创新中迈出新步伐，对外宣传取得新成果。《八公山志》出版发行。启动了承办省第十二届运动会各项筹备工作。人口计生完成省下达的各项考核目标任务。统计、档案、防震减灾取得新进步。妇女儿童、老年人、残疾人权益得到保障，民族、宗教工作取得新进展。外事、对台工作进一步加强，国防动员、人民防空、民兵预备役建设迈上新台阶，创建“全国双拥模范城”实现五连冠。
第七，注重解决民生问题，和谐社会建设基础进一步夯实。投入资金2.3亿元，强力实施十二项民生工程。建立了城乡义务教育“两免一补”保障机制，惠及全市31万中小学生；实施农村饮水安全工程，首批惠及10万人；建立城镇居民基本医疗保障制度，参保人数达到16.8万，扩大农村新型合作医疗范围，参合人数达到76.7万；提高城乡医疗救助水平，建立健全城乡卫生服务体系、重大传染病病人医疗救治和生活救助制度、农村部分计划生育家庭奖励扶助制度；进一步巩固城乡居民最低生活保障制度，完善城镇未参保集体企业退休人员基本生活保障，提高了农村“五保户”供养标准。实行了被征地农民养老保险试点并向全市推开，提高了城镇养老、失业保险标准。全年发放社会保障资金11.5亿元，增长16.6%。新增城镇就业3.2万人，下岗失业人员再就业2.4万人，基本消除了城镇零就业家庭；新增农村劳务输出3.2万人，完成农村劳动力转移培训4543人。实行机关事业单位工资制度改革，提高了全市最低工资标准。开工建设经济适用房32万平方米，发放廉租住房及补贴826户。实施农村扶贫开发整村推进项目13个，加大对山南七个乡镇扶持力度，农村交通、教育等基础设施和生产生活条件得到改善。
第八，推进依法行政，民主法制建设进一步加强。坚持依法行政、依法治市，提请市人大常委会审议地方性法规1件，对政府规章进行了全面清理，修订5件、废止13件，颁布5件，办理行政复议案件27件。“五五”普法深入实施。各级政府自觉接受法律监督、民主监督、舆论监督和社会监督，邀请人大代表、政协委员列席市政府常务会议，全年办理人大代表议案2件、建议78件，办理政协委员提案459件。安全生产、食品药品等领域专项整治和执法监管进一步加强，瓦斯治理走在全国前列，安全生产形势持续平稳。扎实开展平安创建，强力推进公安“三基”工程，社会治安保持稳定，刑事发案率明显回落，人民群众安全感普遍增强。市看守所已建成启用，强制戒毒所和人民警察培训学校基本建成。深入开展矛盾纠纷排查调处，坚持市领导信访接待日制度，一批信访问题得到有效处理。严格落实责任制和责任追究制，强化行政监察和审计监督，惩防并举的反腐倡廉工作力度不断加大。
2007年，是市十三届人民政府任期的最后一年。回顾本届政府任期的五年，面对错综复杂的国际国内发展环境，我们认真贯彻中央和省委、省政府的各项方针政策，抢抓机遇，团结拼搏，攻坚克难，圆满完成“十五”计划，顺利实施“十一五”规划，经济社会发展取得了令人鼓舞的重大成就。这五年，是淮南历史上经济发展最好，改革开放步子迈得最大，城乡面貌变化最快，人民得到实惠最多的时期之一。五年来，经济综合实力显著增强。全市生产总值由155.6亿元增加到355亿元，年均增长14.0%；财政收入增长1.8倍，年均增长23.1%；“三大基地”建设成效显著，一批事关全局的重大工程相继建成，累计完成固定资产投资710亿元。五年来，改革开放不断深化。市场经济体制进一步完善。国有企业改制面达94%；在全省率先实施主辅分离、辅业改制和分离企业办社会职能改革，5.14万国有企业下岗职工出中心工作顺利完成；财税、金融、投资、行政管理等各项改革取得积极进展；累计利用外资7.2亿美元，进出口总额2.9亿美元。五年来，城乡面貌明显改变。山南新区建设全面启动，旧城改造大规模实施；累计竣工城镇住房面积630万平方米，新增城市道路面积150万平方米，绿化覆盖率达40.5%，提高12.7个百分点；一批公用设施相继建成，城市环境质量明显改善；新农村建设扎实推进，城矿乡统筹取得重要进展，城市形象品位显著提高。五年来，社会事业全面进步。各项社会事业市级支出累计30.7亿元，占总支出的55.8%，是前五年的4.4倍；科教兴市战略取得新成效，再次被评为全国科技进步先进市，高中阶段在校生由3.9万人增加到8.3万人；公共卫生体系基本建成，新型农村合作医疗制度覆盖4个县区，参合率达79.6%；人口年自然增长率控制在7‰以内；精神文明和民主法制建设取得新进步。五年来，人民生活水平大幅提高。城镇居民人均可支配收入、农民人均纯收入年均分别增长17.6%和9.2%；累计新增城镇就业岗位15.4万个，下岗失业人员再就业10.3万人；全面实施养老、医疗、失业、工伤、生育保险制度，初步建立城乡居民最低生活保障制度；五年减少贫困人口3.5万人；人民生活正向全面小康迈进。
五年来的成就，是省委、省政府和市委正确领导的结果，是市人大和人大代表、市政协和政协委员及社会各界人士关心支持的结果，是全市人民共同拼搏的结果。在此，我代表市人民政府，向全市人民及驻淮部队、武警官兵、公安干警，向各民主党派、工商联、无党派和各界人士，向关心和支持淮南现代化建设的中央、省驻淮单位及海内外朋友，表示衷心的感谢！
在充分肯定成绩的同时，我们也清醒地认识到，发展不足仍是我市的突出矛盾，我们的工作与科学发展的要求、与全市人民的期望还有不小差距，前进中还面临不少困难和问题，主要是：经济结构不够合理，新兴产业发展滞后，过度依赖资源性产业的格局尚未有效改变，过度依靠投资拉动经济增长的格局没有改变；转变发展方式任务艰巨，经济增长的资源环境成本过高，自主创新能力亟待加强；人均财力、公共服务能力不强，社会事业、城市基础设施历史欠账较多；县区经济、民营经济发展不足，区域之间、城乡之间、行业之间差距拉大；就业再就业和社会保障压力增大，需要解决的民生问题还很多；政府职能转变仍不适应新要求，机关作风和效能建设有待加强，发展环境还不尽如人意；安全生产和社会稳定压力进一步加大。对此，我们必须高度重视，认真加以解决。
二、关于今后工作的总体要求和基本思路
站在新的历史起点上，我们必须进一步强化忧患意识、紧迫意识和责任意识，进一步解放思想，抢抓机遇，创新发展，努力实现经济社会发展的新跨越。总体要求是：高举中国特色社会主义伟大旗帜，以邓小平理论和“三个代表”重要思想为指导，认真贯彻党的十七大精神，全面落实科学发展观，以推动科学发展、构建和谐淮南为主题，以转变经济发展方式为主线，以“9771”提升行动计划为抓手，加快实施工业强市、东向发展、创新推动、城市化带动、合淮同城化、城（矿）乡统筹、可持续发展战略，加速富裕、文明、民主、和谐的现代化进程，把淮南建设成为沿淮城市群中综合实力最���、辐射影响最大的区域中心城市，形成新的皖北“发展极”，向实现全面建设小康社会目标迈出坚实步伐。
我们在充分调研和集思广益的基础上，本着尊重历史、立足现实、面向未来的原则，提出实施“9771”提升行动计划。
——“9”，即重点发展煤炭、电力、化工、机械电子、生物医药、食品加工、新型材料、现代服务、文化九大产业。
——“7”，即继续推进社会保障、就业再就业、教育均衡、全民健康、安居、平安、食品药品放心七大民生工程。
——“7”，即深入实施交通畅通、防洪保安、信息服务、资源节约与环境保护、信用环境、科技创新、人才培养七大基础工程。
——“1”，即实现一大目标：到2020年，人均生产总值比2006年翻两番，达到5.2万元，生产总值年均增长10%以上，达到1400亿元。
未来五年，是深入贯彻落实科学发展观，继续全面建设小康社会的关键阶段，也是大力实施“9771”提升行动计划，加速淮南奋力崛起的关键阶段。要通过五年的不懈努力，到2012年，全市人均生产总值达到2.4万元，工业化率达到55%，城镇化率达到65%，单位生产总值能耗下降15%左右，二氧化硫、化学需氧量排放分别下降20%和11%，城镇居民人均可支配收入和农民人均纯收入分别达到2.1万元和5300元，覆盖城乡的公共服务体系和社会保障体系基本建立，加快形成速度与结构质量效益相协调、经济与社会各项事业发展相协调、城镇化发展与新农村建设相协调、人口资源环境相协调、人人共享发展成果的和谐发展格局。
围绕实施“9771”提升行动计划，实现未来五年发展目标，着力推进“一优化、六统筹”：
优化产业结构。坚持在发展中优化，在优化中提升，做强优势产业，提升传统产业，培育新兴产业。要做精第一产业。着力提高农产品优质化率和转化率，提高养殖业在农业中的比重，提高农业专业化、规模化、产业化水平，促进传统农业向现代农业转变。要做优第二产业。坚持走新型工业化道路，建设高水平、高效益、可持续发展的新型煤电基地和煤化工基地，打造能源重化工业走廊；以交通快速通道为依托，以省级开发区和工业集聚区为载体，推动省级煤机装备制造基地、生物医药及高新技术产业基地和以乳制品、豆制品加工为主的绿色食品基地建设，加快发展新型材料、电子等产业，促进工业集群式、基地化发展。要做大第三产业。重点发展现代金融、现代信息、现代物流、现代旅游、先进文化等产业，打造现代服务业经济增长极，促进生产性服务业和生活性服务业协调发展。要壮大企业主体。进一步发挥骨干企业在产业发展中的支撑带动作用，加强大企业大集团建设，新增一批销售收入超10亿元企业，培育一批“专精特新”的中小企业，形成充满活力的企业梯队。
统筹经济社会各项事业发展。在经济发展的同时，高度重视社会建设，促进经济社会协调发展。深入实施创新推动战略，加快建设以企业为主体、市场为导向、产学研相结合的技术创新体系。优先发展教育事业，整合教育资源，优化教育结构，促进各类教育均衡发展。加快发展卫生事业，完善医疗卫生体系，抓好饮水安全，推进全民健身行动，提高全民健康水平。繁荣文化事业，发展文化产业，建设文化大市。加快建设覆盖城乡居民的社会保障体系，保障人民基本生活，促进城乡充分就业，提高人民群众的生活质量和幸福指数。
统筹人口、资源、环境建设。把人的发展放在首位，全面提高人口素质，深入实施人才强市战略。把建设资源节约型、环境友好型社会放在推进跨越式发展的突出位置，形成有利于可持续发展的经济发展方式和社会生活方式，实现现代化建设与生态文明建设的有机统一。抓住国家促进资源型城市转型的机遇，推进“三废一沉”治理，加快资源枯竭矿区环境修复。抓住国家加大淮河治理的有利时机，推进流域综合治理，变“水患”为“水利”。不断改善城乡生产生活环境，努力使经济社会发展与人口、资源、环境承载能力相适应，增强可持续发展能力。
统筹投资、消费、出口协同拉动。着力解决投资结构不优、消费总量和层次不高、经济外向度偏低的矛盾，使经济增长由主要依靠投资拉动向依靠消费、投资、出口协同拉动转变。积极创新投融资方式，优化投资结构，拓展融资渠道，提高直接融资比重。努力扩大城乡消费，改善消费环境，提高服务业比重和水平。拓展对外开放广度和深度，推动外贸转型升级，增强出口对经济增长的拉动作用，提高开放型经济对经济增长的贡献率。
统筹城矿乡和区域协调发展。围绕实施城市总体规划，按照形成主体功能区的要求，着力提升东部，振兴西部，加快南部开发，促进北部跨越式发展。加快以高速公路为重点的大交通建设，构建城际间、县区间快速通道和综合交通网络，增强产业聚集能力、要素集约能力和辐射带动能力。围绕新农村建设，按照“一整三带”的要求，不断推进农村人口向新型村镇集中，土地向能人大户、规模化种植、集约化经营集中，农业产业化经营向工业园区集中，公共服务资源向人口密集区集中，资金向改善农村生产生活条件集中，着力把县城和重点集镇建设成县区经济增长中心，打造特色鲜明的县区经济板块。支持凤台县向全国百强县目标迈进。围绕市矿统筹，加强采煤沉陷区治理与失地农民保障，推进旧城改造与老矿区修复，协调产业发展与环境保护，努力走出一条城乡统筹、和谐城矿乡的新路子。围绕区域协调发展，实施合淮同城化战略，推进规划同筹、交通同建、通信同城、能源同体、产业同布、市场同构、信息同享、科教同兴、旅游同线、环境同保，逐步实现城市、经济、文化、交通、旅游和环保一体化。
统筹财政、企业、城乡居民收入。在提高发展质量、增加财政收入的基础上，坚持“多予、少取、放活”的方针，加快建立以工促农、以城带乡长效机制，形成规范透明的财政转移支付制度，推进公共服务均等化。着力提高低收入者收入，逐步提高最低工资标准，建立企业职工工资正常增长机制和支付保障机制。创造条件让更多群众拥有财产性收入。保护合法收入，调节过高收入，取缔非法收入，逐步扭转收入分配差距扩大趋势。
统筹改革发展稳定。把最大限度激发社会创造活力与最大限度增加和谐因素有机统一起来，正确处理结构调整与促进就业的关系，正确处理深化改革与维护人民群众利益的关系，正确处理城市建设与居民拆迁安置的关系，正确处理重大项目建设与失地农民保障的关系，以改革促进和谐，以发展巩固和谐，以稳定保障和谐，进一步增强公众安全感、幸福感和社会和谐度。
各位代表，我们正置身于科学发展的伟大时期，肩负着和谐发展的神圣使命。只要我们咬定目标，坚定信心，凝心聚力，破浪前进，就一定能够谱写淮南奋力崛起的新篇章，就一定能够开创全面建设小康社会的新局面！
三、关于2008年主要目标和任务
2008年是新一届政府的开局之年，做好今年的政府工作十分重要。我们要全面贯彻十七大、中央、省经济工作会议和市委八届三次、四次全会精神，深入落实科学发展观，紧紧围绕实施合淮同城化战略和“9771”提升行动计划，坚持好字优先、稳中求进，积极推进改革开放和自主创新，着力优化经济结构和提高经济增长质量，切实加强节能减排和生态环境保护，更加重视改善民生和促进社会和谐，推动经济社会又好又快发展。
主要目标是：生产总值增长14%，财政收入同步增长，固定资产投资180亿元以上，社会消费品零售总额增长12%，城镇居民人均可支配收入增长8%，农民人均纯收入增长6%，新增城镇就业岗位3.2万个，城镇登记失业率控制在4.4%以内，人口自然增长率控制在7.5‰以内，单位生产总值能耗降低4.4%，二氧化硫、化学需氧量排放分别下降1%和3%，亿元生产总值生产事故死亡率下降9.59%。为实现上述目标，着重抓好九个方面工作：
（一）突出工业强市，促进产业结构优化升级
强力推进优势产业规模发展。要坚定不移地支持煤电化三大传统产业的技术化改造、基地化建设、规模化发展和信息化提升。开工建设杨村、朱集等煤矿，确保凤台电厂一期建成发电，全面建成500千伏西通道工程淮南段、潘谢矿区铁路等配套项目，加快形成“亿吨煤基地”和“千万千瓦火电基地”；开工建设煤化工新区及淮化60万吨甲醇、20万吨二甲醚项目，加快甲醇制丙烯工业试验装置、淮南矿业集团煤制焦油、德邦100万吨纯碱扩建二期、蓝天化工新厂等项目建设，促进淮化合成氨老系统改造、煤基合成油催化剂等项目建成投产，推动安徽煤化工基地取得突破性进展。要坚定不移地促进具有后发和持久优势的产业在经济总量中占有更多的份额、更大的比重。做大做强凯盛重工、舜立机械、长壁煤矿机械，促进淮南矿业集团与美国比赛洛斯公司的合作，全面振兴省级煤机装备制造基地；积极整合药业资源，培育药业拳头产品，塑造药业品牌，不断壮大省级生物医药及高新技术产业基地；加快钢铁、纺织、服装等产业改造升级，扩大汽车配件、印刷机械、矿用电子、光纤光缆等产业发展规模；充分发挥益益乳业、八公山豆腐行业协会等龙头企业带动作用，提高食品加工业集中度。着力抓好56个投资千万元以上技改项目，力争新增规模以上工业企业20户。
强力推进开发区和工业园区产业集聚。集中项目、集中力量支持开发区加快发展，完善基础设施和物流配送、劳动用工、信息咨询等产业配套，全面提升园区产业承载力和集聚力。重点抓好力达电器等11个项目开工，促进景丰万吨高档卷烟纸、益益万吨酸奶等10个项目年内竣工投产，完善开发区集中供热系统，开工建设中兴路，基本建成规划路、田东路。加快县区工业化进程，着力推进凤台开发区、淮南工业园和县区工业集聚区建设，使园区成为县区工业发展的主引擎。
强力推进服务业现代化。加快培育新的消费热点，积极发展与消费升级相关的产业。加快引进大企业、大集团和战略投资者，实施旅游“4461”工程，进一步完善旅游基础设施和配套建设。加快八公山、上窑山、卧龙山、茅仙洞等生态文化旅游开发，加快焦岗湖、高塘湖、城北湖等休闲度假旅游开发，加快近郊、沿湖农家乐旅游和工矿旅游开发。全力做好中国优秀旅游城市达标验收的准备工作。积极发展现代物流，重点抓好综合物流园区、物流配送中心和物流信息平台建设，开工建设医药物流园。大力发展现代金融、信息服务等产业。改造提升商业贸易、交通运输、餐饮娱乐等传统服务业，发展特许经营、连锁经营、加盟经营等新型业态，抓好家乐福购物广场、商之都淮南购物中心、财富中心、古井瑞景国际购物中心建设，提高特色商业街档次和水平。继续推进农贸市场升级改造和“万村千乡”市场工程，促进城乡商品流通无缝对接。完善房地产市场体系，健康发展房地产业，全年开工住宅面积180万平方米，竣工120万平方米。
强力推进重大项目建设。坚持把重大项目作为产业结构调整的重要抓手，围绕实施“9771”提升行动计划，培育项目主体，提升项目质量，优化项目结构，力争全年实施投资千万元以上项目220个，其中亿元以上项目58个，同比分别增加38个和15个。
（二）实施创新推动，加快转变发展方式
更加注重科技创新。推动创新企业示范，力争认定高新技术企业3家、高新技术产品2项，实现民营科技企业技工贸收入20亿元。实施创新项目带动，力争列入国家火炬计划3项、创新基金项目6项。抓好创新平台建设，着力推进淮南煤直接气化等重大科技攻关，加快瓦斯治理国家工程研究中心、高新技术创业服务中心建设，筹建国家级煤化工产品监督检验中心和安徽省药用辅料工程技术研究中心，深化市校合作，促进科技成果向现实生产力转化。实施“数字淮南”工程。整合创新资源，发挥各类优惠政策的导向和激励作用，优化创新环境，集聚创新人才，使全社会创新智慧竞相迸发，各方面创新人才大量涌现。
更加注重节能减排。加强煤炭、电力、化工、钢铁、建材等重点行业整治，对纳入监控重点的17户耗能企业和14户污染企业，实施主要产品能耗标准管理，完成8个城市污水处理及工业废水治理、5个二氧化硫综合治理项目，确保污染物排放总量控制。坚持“关小”与“上大”同步实施，淘汰落后与技术改造相结合，引导帮助拟淘汰企业转产、技改或新上技术先进的产业项目。
更加注重循环经济发展。加快发展以煤热解为核心的热、电、气、焦油多联产系统，推动淮南矿业集团煤焦油深加工、乙炔化工等项目建设；加快发展以煤气化为核心的煤基多联产系统，推动淮化老厂区循环型改扩建，建设循环型煤化工新区；加快发展以工业废弃物、农作物秸秆为主的能源资源综合利用系统，推动与香江集团合作建设煤电铝循环经济工业园，促进顾桥、新庄孜等煤矸石电厂加快建设，新上一批低温余热发电、农作物秸杆发电和煤矿瓦斯综合利用项目，鼓励企业利用煤矸石、粉煤灰和石膏发展新型建材。
更加注重生态建设。继续做好国家卫生城市、国家环保模范城市创建工作，抓好国家园林城市、生态省建设综合示范试点市建设，新增城市园林绿地100公顷，新建街头游园8处，完成成片造林1.7万亩。全面启动舜耕山风景区核心区、淮河公园、淮西湖公园建设和龙湖公园改造，加快泉大资源枯竭矿区环境修复开发、十涧湖国家城市湿地公园、龙泉公园建设。推进采煤沉陷区综合治理，统筹做好农民搬迁、新村镇建设、沉陷区修复、失地农民保障等工作。强化山石资源和自然植被保护，推进土地、矿产资源节约集约高效利用，落实耕地占补平衡措施，整理复垦土地540公顷。
（三）强化“三农”工作，扎实推进社会主义新农村建设
加快推进农业产业化。深入实施小麦优质高产攻关、稻米产业提升行动和粮食丰产科技工程，加快蔬菜、瓜果、大豆、花卉、苗木等高效产业带建设。支持规模养殖业示范基地建设，认真落实生猪和奶牛生产扶持政策，继续实施奶牛扩群工程，力争养殖业产值占农业总产值45%以上，提高2个百分点。重点扶持焦岗湖斑点叉尾？扩繁、南美白对虾规模养殖、八公山豆制品加工新区、顾桥面粉集团、夏集宏宇集团、佳益米业万吨米糠油加工、兴杨公司2千万只肉鸡标准化养殖区等千万元以上龙头项目，实现市级龙头企业总产值增长10%以上。争创无公害农产品、绿色食品、省级以上名优农产品10个，力争农产品优质率达75%，提高5个百分点。推进科技兴农，争创2个省级农业科技示范园区，引进20个新品种、推广15项新技术。加强农业标准体系和农产品质量检测体系建设，完善农产品安全监管和动植物病虫害防控体系。
加快农村基础设施建设。加大强农惠农政策力度，争取国家和省对治淮工程、扶贫开发、江淮分水岭治理、灾后重建的支持，着力抓好农村道路、通讯广播、泵站修复、农田水利、行蓄洪区移民迁建等重点建设。启动淮河石姚段、洛河洼行洪区改造，争取实施焦岗湖、西淝河、架河低洼地治理，完成淮北大堤加固、行洪区堵口复堤等工程建设。新建“乡乡通”水泥路70公里，完成“村村通”延伸工程190公里，基本实现村村通班车，建成养沼一体化大型沼气池5个、小型池2000个，新建农家书屋140个。
加快农村城镇化。完善乡镇规划、村庄布点规划，深入实施新农村建设“1860”示范工程，启动平圩、上窑、夏集等小城镇改造，完善曹庵、朱马店、顾桥、桂集等小城镇功能，推进采煤沉陷区移民安置新区和新型村落社区建设。全面实施农村生态家园工程，推进“一绿两清五改”，美化村容村貌，净化人居环境。
加快培育新型农民。继续实施农民工转移培训工程，提高农民科技文化素质，鼓励发展中介服务组织，提高劳务输出的组织化程度。着力抓好“一村一名大学生”计划，全年完成各类农民培训6万人。
（四）深化改革开放，激发全民创业活力
着力推进市场化取向改革。深化企业改革，抓好17户国有企业改制重组，完成10户市属出中心企业产权置换，支持淮南矿业集团、淮化集团上市。加强国有资产监管和保值增值，抓好全市不良金融资产分包处置工作。深化财税、金融体制改革，进一步完善公共财政制度，强化税收征管，着力发展地方性银行机构。加强政银、银企合作，完善各类担保体系建设，提升各类融资主体融资功能。深化农村综合改革，积极探索农村土地有效流转，基本完成集体林权制度改革。扎实推进“信用淮南”建设，规范发展行业协会和市场中介组织，健全社会信用体系。深入整顿规范市场秩序，加强食品药品市场监管，严厉打击制假售假、商业欺诈、坑民害民等违法行为。推进质量兴市，强化名牌战略。发展各类要素市场，加快形成统一开放、竞争有序的现代市场体系。
着力提高开放型经济和民营经济水平。加强周边区域合作，加深东向产业对接，加快经济国际化进程，加速形成内外联动、纵深推进的开放格局。拓宽招商的广度和深度，增强引资的针对性和选择性，变招商引资为招商选资，变政策招商为环境招商，吸引一批国内外战略投资者特别是中央直属大企业、大集团来淮南投资，主攻一批对实施“9771”提升行动计划有牵动性的大项目、好项目，力争全年利用外资1亿美元，引进内资100亿元。鼓励和支持企业扩大外贸出口，力争全年完成进出口7000万美元。深入推动全民创业，浓厚创业氛围，优化创业环境，着力完善民营、中小企业信息网络、创业培训、信贷融资、知识产权等方面的服务体系。建立5个创业基地，培植30个民营项目，促进30户民营企业上规模、上水平。
（五）推进合淮同城化，扩大城市辐射力和影响力
大力拓展区域合作领域和空间。坚持区域经济一体化发展方向，在巩固提高“两淮一蚌”城市群协作发展的同时，重点实施南向突围战略，积极构建合淮新型区域经济联合体，加速融入省会经济圈。推进规划先行，重点抓好合淮同城化总体规划、区域城乡一体化规划、大交通及能源规划、工业走廊规划、生态规划的制定和实施；推进基础设施深度对接，开工建设淮蚌高速公路淮南段、合淮蚌铁路客运专线、阜淮铁路和淮南铁路合淮段电气化改造、淮河二桥、206国道改线工程，做好淮滁高速公路、城市外环路、凤台二桥的前期工作，确保合淮阜高速公路全线建成通车；推进现代产业互动发展，启动合淮工业走廊建设；推进合作机制加快形成，建立高层定期协商机制和专项联系会议制度，设立日常工作机构，在破解瓶颈制约方面积极探索，在促进区域合作方面率先突破。
大力完善城市布局和功能。研究制定我市主体功能区规划，强化对区域发展的引导和规范。深入开展主城区30平方公里控制性详细规划编制；继续推进主城区地下管网普查、城市重要地段和节点设计；完成西部城区、潘集区等分区规划编制。加快山南新区开发。开工建设起步区15平方公里路网、水系景观、农民新村以及市行政中心、山南自来水厂、新集企业会所、移动公司生产楼、武警支队营房等进区项目；确保洞山隧道、陈洞南路（至合淮阜高速公路）建成通车；完成山南高压走廊改造工程。加快城市基础设施建设。启动东西部第二通道建设；开工建设国庆东路、淮舜南路、夏郢孜西路和医疗废弃物处置中心；加快建设天然气城市利用、城市集中供热改造、公交IC卡无人售票、东部生活垃圾处理场、洛河1号排污沟整治等工程；完成洞山西路、龙湖路、淮河大桥、凤台大桥等改造；建成湖滨东路；逐步实施龙王沟、洞化截洪沟、八公山截洪沟水系改造。加快老城区和城中村改造。继续推进23个已批旧城改造项目，改造小街小巷30条。
大力强化城市经营和管理。进一步完善土地收储和经营机制，吸引外资、民资进入城市道路、桥梁、园林绿化、污水处理、集中供热等市政公用行业，加快形成多元化的城市建设投融资机制。推进城市管理重心下移，强化城市综合执法，积极推行建管分开、管养分离和委托承包等方式。深入开展文明城区、文明社区、文明小区、文明行业创建活动。强化“门前三包”责任，落实“四定”管理，形成高效保洁网络。加强中巴车、出租车、三轮车、户外广告整治，规范流动摊点、农贸市场、小区物业管理，坚决遏制城乡违法建设。
（六）建设文化大市，增强淮南发展的软实力和凝聚力
促进文化事业大繁荣。坚持开发与保护并举，打好淮南子文化、花鼓灯文化、豆腐文化、少儿艺术和旅游文化“五张牌”。推进文化内容、形式、载体、传播手段和体制机制创新，创作更多体现时代精神、群众喜闻乐见、具有淮南特色的优秀艺术作品。做好科技馆前期工作，开工建设八公山地质博物馆，加快少儿艺术、花鼓灯艺术学校及研发基地建设，全面完成博物馆二期续建，正式开放山南新区规划展览馆。推进农村广播电视、综合文化站等服务项目建设，着力构建公共文化服务体系，保障人民群众基本文化权益。精心办好第十五届中国豆腐文化节、首届中国民间舞蹈艺术节暨第二届淮河花鼓灯艺术节。
促进文化产业大发展。深化文化体制改革，实施文化产业项目带动、品牌推进和外向拓展战略，采取特色化引领、基地化开发的形式，建设一批文化产业基地、文化产业园，打造城市文化产业功能区。健全文化市场体系，活跃文化市场主体，引导文化消费，培育文化业态。大力发展新闻出版、演艺娱乐业，促进大型节庆活动、旅游纪念品、艺术创作、网络服务等产业化发展。
（七）大力改善民生，努力构建和谐社会
以公共服务均等化为目标，加快发展社会事业。强化政府对义务教育的保障责任，改善农村中小学办学条件，加快中小学布局调整，促进义务教育均衡发展。提高高中教育质量，高中阶段教育毛入学率达到69.1%。大力发展职业教育，加快淮南技工学校新校区建设。支持高等教育快速发展。坚持公共医疗卫生的公益性质，建立健全覆盖城乡居民的公共卫生服务体系、医疗服务体系、医疗保障体系、药品供应保障体系。加快市第一人民医院外科大楼、马山医院新病区建设。突出抓好艾滋病、结核病、乙肝等疾病防治，启动县区农村卫生监督体系试点建设。广泛开展“全民健身与奥运同行”活动，确保“奥运火炬传递”顺利进行。切实做好承办省第十二届运动会和全省第二届体育大会各项筹备工作，加快市水上运动中心、全民健身综合训练馆、凤台体育中心等场馆建设。加强人口和计划生育工作，稳定低生育水平，提高人口素质，努力降低出生人口性别比。加强民族宗教、外事侨务和人民防空工作。
以创业带动就业为抓手，大力促进就业再就业。加快实施就业扶助工程，统筹做好失地农民、城镇下岗失业人员、高校毕业生、复转军人就业工作，全年实现下岗失业人员再就业1万人，新增农村劳动力转移3万人。完善公共就业服务体系，加强就业和创业技能培训，建立统一规范的劳动力资源市场。规范和协调劳动关系，依法保护劳动者合法权益。
以深入实施民生工程为重点，继续完善社会保障体系。认真落实省政府十八项民生工程，加大投入，严格标准，完善措施，让广大人民群众得到更多的实惠。加快建立覆盖城乡居民的社会保障体系，积极推行“五保合一”，实行失业保险市级统筹。扩大被征地农民养老保险范围，实现新型农村合作医疗、城镇居民医疗保险全覆盖。大力发展慈善事业和社会福利事业，完成10所农村敬老院改造。加快安居工程建设，开工建设经济适用房30万平方米、廉租房1000套。加强价格监管，认真落实“菜篮子”工程一把手负责制，切实保障人民生活需求。
（八）完善社会管理，维护社会安全稳定
加强民主法治和思想道德建设，巩固社会和谐基础。完善基层民主，做好第七届村民委员会换届选举工作。全面实施“五五”普法，加强司法工作，发挥公证、仲裁、人民调解作用，维护社会公平正义。以建设社会主义核心价值体系为引领，加强社会公德、职业道德、家庭美德、个人品德建设，提高公民思想道德素质。深入开展国防动员教育和双拥共建活动，巩固军政军民团结。
加强平安淮南建设，保持社会安定有序。深入开展社会治安综合治理，健全防控体系，扎实推进公安“三基”工程，严厉打击刑事犯罪、经济犯罪活动，努力打造全省最安全的城市，切实保障人民生命财产安全。认真落实信访稳定责任制，畅通信访渠道，努力实现信访减量退位。坚持安全发展，强化安全生产监管，全面排查和治理各类安全隐患，有效防止重特大安全事故发生。完善应急管理机制，提高应对突发公共事件的能力。
（九）加强政府自身建设，提高科学发展能力
要努力建设法治政府。切实增强科学发展的法治观念，把依法行政贯穿于政府决策、执行、监督的全过程，严格依照法定权限和程序行使权力、履行职责。依法接受人大及其常委会的法律监督，主动接受政协的民主监督，自觉接受社会各界和人民群众的监督。围绕“9771”提升行动计划，按照法定程序修编“十一五”规划。加强对各级行政机关及其工作人员依法行政的教育和培训，着力解决企业和群众反映强烈的多头执法、执法扰民等问题。
要努力建设服务政府。适应体制转轨、社会转型的新变化，推动政府职能由“管、审、批”向“扶、帮、助”转变，把工作切入点体现到科学发展、和谐发展上来，把工作着力点放到主要为经济社会发展创造良好环境上来，把工作落脚点集中到解决民生问题上来。健全部门之间的协调配合机制，优化政府部门职责分工。深化行政审批制度改革，精简行政审批事项，继续推进向县区放权，进一步释放行政管理权限的空间和活力。
要努力建设透明政府。深入推进办事公开，加强电子政务，拓展网上审批和服务范围，提高在线办事水平。完善市、县区、乡镇三级政务服务中心建设，形成上下联动的审批绿色通道。健全公众参与、群众监督和政府决策相结合的行政决策机制，推行政务发布、听证论证、通报质询等形式，办好市民互动系列栏目，努力使政府的各项决策合民意、顺民心。
要努力建设责任政府。深入开展效能建设，建立科学发展的目标考核体系，进一步落实科学发展的责任制和责任追究制，坚决防止上行下不通、上决下不定的问题，坚决杜绝推诿扯皮、各行其是的现象，坚决查处不作为、乱作为和失职渎职行为，切实做到责任主体明确、责任层级清晰、责任链接无缝，确保各项目标任务落到实处。
要努力建设廉洁政府。坚持标本兼治、综合治理、惩防并举、注重预防的方针，扎实推进惩治和预防腐败体系建设，拓展从源头上防治腐败的工作领域，坚决防止滥用权力的谋私行为，坚决纠正损害群众利益的不正之风，坚决惩治各种形式的腐败分子，确保政务规范，政事清明，政风廉洁。
各位代表，新的发展蓝图，令人鼓舞；新的形势任务，催人奋进。让我们在中共淮南市委的领导下，紧密团结在以胡锦涛同志为总书记的党中央周围，高举中国特色社会主义伟大旗帜，以十七大精神统领经济社会发展全局，锐意进取，励精图治，为夺取全面建设小康社会新胜利而努力奋斗！

附件：

特定词语注释

1、六有一无：即有饭吃、有地方住、有衣穿、有干净水喝、有病能医、有干部管理服务，无大的疫情发生。
2、5+1：“５”即少儿舞蹈、声乐、器乐、书法、绘画五项比赛，“１”为全国少儿产品博览会。
3、公安“三基”工程：即公安机关开展的抓基层、打基础、苦练基本功工程。
4、一整三带：市委八届四次全会提出，按照“一整三带”的思路，搞好土地规整，规划建设一批相对集中、设施完善的农民新居，带动新村镇建设；规划建设与新村镇相配套的产业园区，让住上新房的农民有事可做，带动产业持续发展、农民富裕安康；加强对农民的业务指导和技能培训，带动素质提高。
5、三废一沉：即废气、废水、废渣和采煤沉陷区。
6、旅游“4461”工程：即依托自然山水、历史文化、工业旅游、道教和佛教4大板块旅游资源，打造八公山、上窑山、焦岗湖和茅仙洞4大精品景区，推出6条精品旅游线路，实现把淮南建成皖西北旅游中心1大目标。
7、新农村建设“1860”示范工程：即建设1个示范区、8个示范乡镇、60个示范村。
8、一绿两清五改：即绿化村庄，清垃圾、清路障，改水、改厕、改圈、改灶、改居。
9、门前三包：即包卫生、包秩序、包绿化。
10、“四定”管理：即实行定人、定时、定岗、定责管理。
11、省政府十八项民生工程：全省经济工作会议决定，在继续实施12项民生工程的基础上，2008年再增加6项。即：建立农村居民最低生活保障制度、进一步提高农村“五保户”供养标准、完善城镇未参保集体企业退休人员基本生活费保障机制、积极推进农村新型合作医疗制度、探索建立城镇居民基本医疗保障制度、逐步提高城乡医疗救助水平、逐步建立重大传染病病人医疗救治和生活救助保障机制、积极推进城乡卫生服务体系建设、全面实施城乡义务教育经费保障机制改革、全面消除农村中小学危房、加速推进农村饮水安全工程建设、进一步完善农村部分计划生育家庭奖励扶助制度等12项，新增加广播电视村村通、高校和中职学校学生资助、大中型水库移民后期扶持、农村公路村村通、解决城市低收入家庭住房困难及贫困白内障患者复明等6项。
12、五保合一：即实行养老、医疗、失业、工伤、生育等五种社会保险统一办理。
